# Assessing the Effects of Dietary Cadmium Exposure on the Gastrointestinal Tract of Beef Cattle via Microbiota and Transcriptome Profile

**DOI:** 10.3390/ani13193104

**Published:** 2023-10-05

**Authors:** Xinxin Xu, Zebang Xu, Bin Yang, Kangle Yi, Fang He, Ao Sun, Jianbo Li, Yang Luo, Jiakun Wang

**Affiliations:** 1 Institute of Dairy Science, College of Animal Sciences, Zhejiang University, Hangzhou 310058, China; xinxinxu@zju.edu.cn (X.X.); zebangxu@zju.edu.cn (Z.X.); rayyangbin@163.com (B.Y.); 2 School of Biological and Chemical Engineering, Zhejiang University of Science and Technology, Hangzhou 310023, China; 3 Hunan Institute of Animal and Veterinary Science, Changsha 410131, China; yikangle@yeah.net (K.Y.); hf3893@126.com (F.H.); kkkkanty@163.com (A.S.); ljbljb12@126.com (J.L.)

**Keywords:** Cd, cattle, rumen, jejunum, colon, microbiota, transcriptome

## Abstract

**Simple Summary:**

Cadmium is a ubiquitous environmental pollutant present in soil, which can be absorbed by plants, animals, and humans, causing tissue damage or dysfunction. Ruminants possess rumen microorganisms to establish tolerance against plant toxins. However, there are few studies describing the response of ruminants to cadmium accumulated in plants. The objective of this study was to evaluate the impact of feeding cadmium-accumulated maize on growth, gastrointestinal response, and microbial alterations in cattle. The results show that cadmium-accumulated maize promoted weight gain and feed efficiency and increased the abundance of short-chain fatty acid-related bacteria, but also had suppressive effects on gastrointestinal immune regulation at the genetic level.

**Abstract:**

Cadmium (Cd) is an environmental pollutant, widely existing in soil, and can be absorbed and accumulated by plants. Hunan Province exhibits the worst cadmium contamination of farmland in China. Ruminants possess an abundant microbial population in the rumen, which enables them to tolerate various poisonous plants. To investigate whether the rumen microbiota could respond to Cd and mitigate the toxicity of Cd-accumulated maize to ruminants, 6-month-old cattle were fed with 85.82% (fresh basis) normal whole-plant maize silage diet (CON, *n* = 10) or Cd-accumulated whole-plant maize silage diet (CAM, *n* = 10) for 107 days. When compared to the CON cattle, CAM cattle showed significantly higher gain-to-feed ratio and an increased total bacterial population in the rumen, but a decreased total bacterial population in the colon. CAM cattle had higher relative abundance of *Prevotella* and *Lachnospiraceae ND3007* group in the rumen, and *Lachnospiraceae NK4A136* group and *Clostridia vadinBB60* group in the colon. Notably, microbial correlations were enhanced in all segments of CAM cattle, especially *Peptostreptococcaceae* in the jejunum. Transcriptome analysis revealed down-regulation of several immune-related genes in the rumen of CAM cattle, and differentially expressed genes in the rumen were mostly involved in immune regulation. These findings indicated that feeding Cd-accumulated maize diet with a Cd concentration of 6.74 mg/kg dry matter (DM) could stimulate SCFA-related bacteria in the rumen, induce hormesis to promote weight gain, and improve energy utilization of cattle.

## 1. Introduction

Cadmium (Cd), an environmental pollutant, is classified as a Group 1 carcinogen by the International Agency for Research on Cancer [1] and is ubiquitous in nature [2]. For nonsmokers, the main sources of Cd toxicity are diet and contaminated water [3], making the gastrointestinal tract the first body part to be affected by Cd toxicity. Yang et al. [4] found that broilers that received a basal diet with 4 mg/kg CdCl2 (dry matter, DM basis) exhibited jejunal villi fusion, necrosis, exfoliation, and inflammatory cell infiltration of the surface epithelium, and Cd also decreased levels of genes related to the intestinal barrier and inflammation in the jejunum. Similarly, Xie et al. [5] reported that mice showed intestinal mucosal barrier damage and inflammation in the intestine after oral administration of 12.5 mg/kg body weight of CdCl2. Furthermore, Chen et al. [6] demonstrated that swine fed a diet containing 20 mg/kg CdCl2 showed necroptosis and inflammation in the small intestine due to the accumulation of reactive oxygen species (ROS) and imbalance of Th1/Th2, respectively.

The toxic effects of Cd are not limited to gastrointestinal damage and inflammation, and Cd can also have systemic impacts on the body through gut microbiota [7]. Zhang et al. [7] found that exposure of mice to 10 mg/L Cd for 10 weeks resulted in a significant decrease in bacteria associated with decompensated cirrhosis and an increase in bacteria related to lipopolysaccharide (LPS) at the family level in the cecum, and these alterations in gut microbiota were consistent with an increase in triglycerides (TG) in the liver, as well as the elevated levels of LPS, free fatty acid (FFA), and TG in the blood serum. Additionally, the mRNA expression levels of key genes involved in FFA synthesis and transport and TG synthesis in the liver were also increased under Cd exposure. Ba et al. [2] demonstrated that administration of 100 nmol/L Cd continuously to the neonatal mice (parents received 100 nmol/L Cd at 1 week before mating) reduced microbial diversity and the abundance of *Bifidobacterium* and *Prevotella* in feces at the eighth week, and these alterations in microbiota contribute to adiposity in adult male mice.

As described above, numerous studies have investigated the toxicity of Cd in monogastric animals. However, research on the effects of Cd on the gastrointestinal tract of ruminants and their microbiota are still lacking. Ruminants possess a more complex digestive system compared to monogastric animals. The rumen in ruminants is directly exposed to ingested feed rather than residual food after digestion, which allows for prolonged interactions between the ingested feed and rumen microorganisms. Due to long-term and continuous exposure to toxic substances, rumen microbial populations can adapt and detoxify phytotoxins, enhancing the tolerance of poisonous plants in ruminants, which has been confirmed [8]. Soil cadmium can be absorbed and accumulated by maize [9], and simultaneously, whole-plant maize silage is a high-quality feedstuff that is widely used in cattle diet [10]. Considering the ability of rumen microbiota to tolerate toxic plants and their diverse functions in the rumen, we hypothesized that the gastrointestinal microbiota might play an important role in responding to and mitigating the potential adverse effects of Cd-accumulated maize in ruminants. Therefore, we aimed to investigate the response of the gastrointestinal tract of ruminants, particularly the rumen, to the diet containing Cd-accumulated maize.

## 2. Materials and Methods

### 2.1. Animals, Feeds, and Experimental Design

All experimental protocols and procedures used in this study were conducted with the approval of the Animal Care and Use Committee of Zhejiang University (Hangzhou, China) (ZJU20230094).

This experiment was conducted for 107 days on a farm located in Huayuan County, Hunan Province, China. Twenty healthy 6-month-old weaned crossbred cattle (Angus × Xiangxi, males and females in equal proportion, initial body weight = 78.50 ± 18.45 kg, mean ± standard deviation) were selected and randomly divided into two groups with equal numbers of males and females: the control group (CON) and the Cd-accumulated maize group (CAM). All cattle were fed with a total mixed ration consisting of corn stover (2.84% fresh basis), whole-plant maize silage (85.82% fresh basis, to test the health risks of Cd-accumulated maize in cattle, we maximized the whole-plant maize silage in the diet of growing beef cattle), and pelleted concentrate (11.34%) (Table 1) at 07:00 and 17:00, and water was available ad libitum throughout the experiment. The whole-plant maize silage was the sole source of Cd, and the Cd concentrations in diet were consistent during the animal trial. The silage used for CON was made from the whole-plant maize planted in Xiangxi Tujia and Miao Autonomous Prefecture, Hunan Province, China, where the Cd concentration in the soil met the requirements of the standard for agricultural soil in China (GB 15618-2018, Cd concentration ≤0.3 mg/kg, pH ≤7.5). The silage used for CAM was made from the whole-plant maize harvested from soils with a Cd concentration of 4.76 mg/kg in Liuyang City, Hunan Province, China. The Cd concentrations of the CON and CAM diets were 0.72 and 6.74 mg/kg DM, respectively, and the Cd concentration in diets of each group were consistent with the initial concentration throughout the animal experiment. Cattle in each group were housed in a pen (4 m × 7.5 m × 1.5 m), separated by railings according to gender. Compared to previous studies [6,11,12], the maximum Cd consumption in our study was approximately 0.28 mg/kg BW (based on DMI and initial BW) at the beginning of the animal trial, which was much lower than these studies.

One male cattle from the CAM exhibited growth arrest, and as a result, the data of this cattle were removed from subsequent analysis.

### 2.2. Growth Performance, Morphology, Serum Parameters, and Cadmium Accumulation

The performance measurement, morphological observation, Cd analysis, and serum parameters determination have been reported in a previous study [13]. Briefly, feed intake was measured every 15 days, and BW was measured at the beginning (Day 0) and at the end (Day 107) of the experiment. On Day 107, all cattle were slaughtered using electrical stunning and exsanguination after fasting for 24 h. Content samples from the rumen, jejunum, and colon were collected and stored in 2 mL centrifuge tube at −20 °C. A portion of tissues samples from the rumen, jejunum, and colon were collected and fixed in formaldehyde solution (10%) for morphological observation, and the other tissue samples were stored at −80 °C for RNA extraction. Blood samples from jugular vein were collected on Day 0, 40, 70, and 107, and the serum samples were stored at −80 °C.

Specimens of rumen, jejunum, and colon tissue were dehydrated and embedded in paraffin, sectioned (5 μm), stained with hematoxylin and eosin (HE), and observed under an optional microscope (80i, Nikon, Tokyo, Japan).

The levels of serum parameters have been reported in our previous study [13], and data were re-used for WGCNA. The concentrations of blood urea nitrogen (BUN), glucose (GLU), creatinine (CREA), uric acid (UA), triglycerides (TG), cholesterol (CHOL), high-density lipoprotein cholesterol (HDL-C), low-density lipoprotein cholesterol (LDL-C), and serum amyloid-A (SAA) were determined with commercial kits (Nanjing Jiancheng Bioengineering Institute, Nanjing, China) using the Hitachi 7020 autobiochemistry instrument (Hitachi, Tokyo, Japan). The concentrations of immunoglobulin A (IgA), IgG, IgM, complement 3 (C3), C4, lipopolysaccharide (LPS), interleukin-1β (IL-1β), IL-6, IL-22, interferon-γ (IFN-γ), tumor necrosis factor-α (TNF-α), and insulin growth factor-1 (IGF-1) were measured with commercial ELISA kits (mlbio, Shanghai, China) using a microplate reader (RT-6100, Rayto, Shenzhen, China).

Samples of blood, content, and tissue from rumen, jejunum, and colon were used to determine the accumulation of Cd. Approximately 1 g (content samples and tissue samples) or 1 mL (blood samples) sample was digested with 3 mL of nitric acid. Then, all solutions were diluted to 10 mL with 5% HCl. The Cd concentration was determined with inductive coupled plasma-mass spectrometry (7700x, Agilent Technologies Inc., Santa Clara, CA, USA).

### 2.3. DNA Extraction and Real-Time PCR

The total DNA of gastrointestinal content samples was extracted using the CTAB (cetyltrimethylammonium bromide) method [14] with a bead-beater (Biospec Products; Bartlesville, OK, USA). The quality and concentration of the extracted DNA were evaluated using 1% agarose electrophoresis and a NanoDrop 2000 spectrophotometer (Thermo Scientific, Waltham, MA, USA). The abundance of total bacteria in each sample was quantified using the ABI 7500 real-time PCR system (Applied Biosystems, Foster, CA, USA) with the primer set from Nadkarni et al. [15] and was calculated as the copy numbers of the 16S rRNA gene per gram of wet content.

### 2.4. Amplicon Sequencing

DNA samples were amplified with the primer set 338F/806R to obtain amplicon libraries of the V3-V4 hypervariable region of bacterial 16S rRNA genes. The amplicon libraries were pooled at an equal molar ratio and prepared for sequencing on the Illumina MiSeq platform by Personal Biotechnology Co. (Nanjing, China) to obtain 2 × 300 bp paired-end reads.

### 2.5. Metataxonomic Data Processing and Analysis

The raw data were trimmed to remove barcodes and residual primers using Cutadapt [16]. Sequences with N’s and Q < 20 were filtered out using Vsearch [17]. Then, the clean data were processed with the DADA2 plug-in for denoising in QIIME2 (version 2022.2) [18]. After merging the paired reads and filtering chimera, an amplicon sequence variant (ASV) table was constructed. Then, taxonomic assignments of ASVs were performed using the SILVA 16S rRNA gene (version 138) database [19]. To analyze the alpha and beta diversity, the sequences of each sample were rarefied to the same size with the minimum number of sequences in samples (18339 sequences/sample). ASVs observed in at least 20% of cattle in every individual group were subjected to downstream analysis.

To identify the ASVs that were potentially associated with Cd-accumulated maize ingestion, linear discriminant analysis effect size (LEfSe) was conducted using LEfSe (http://huttenhower.sph.harvard.edu/galaxy/) (accessed on 17 October 2022). Additionally, correlations among ASVs were analyzed using the SparCC algorithm [20] based on their counts. Correlations with *p* < 0.05 and |coefficient R| > 0.75 were visualized using Cytoscape (version 3.8.2).

### 2.6. RNA Isolation and Transcriptome Data Processing and Analysis

The total RNA from each gastrointestinal tissue was extracted using a total RNA extraction kit (Aidlab, Beijing, China). The RNA quality was evaluated using an Agilent 2100 bioanalyzer (Agilent Technologies, Santa Clara, CA, USA). Only RNA samples with integrity greater than 7 were included in the subsequent RNA sequencing library construction. Following this criterion, three samples with low integrity (two from the CON and one from CAM in the colon) were excluded from subsequent sequencing. RNA sequencing was performed using the Illumina NovaSeq platform by Personal Biotechnology Co. (Nanjing, China) to generate 2 × 250 bp paired-end reads. Low-quality reads (base quality < Q20 bases) and adapters were discarded. Salmon (version 1.5.1) [21] was used to align the high-quality reads to the bovine genome (http://www.ensembl.org/index.html, ARS-UCD1.2) (accessed on 29 November 2022) to determine transcripts per million (TPM) in individual biological samples.

Differentially expressed genes (DEGs) between CON and CAM were identified using DESeq2 package [22] in R, with |log_2_fold change| > 1 and *FDR* < 0.05 (rumen) or *p* < 0.05 (jejunum and colon). DEGs were annotated based on the Gene Ontology (GO) database using Database for Annotation, Visualization, and Integrated Discovery (DAVID). GO terms with *p* < 0.05 were considered significantly enriched and were clustered using simplyEnrichment R package [23].

The function varianceStablizingTransformation in the DESeq2 package was used to normalize the read counts of genes for Weighted Gene Coexpression Network Analysis (WGCNA) [24]. The blockwiseModules function (minModuleSize = 30, merCutHeight = 0.2, and deepSplit = 1) was used to generate gene coexpression modules. Subsequently, correlations between the modules and traits (growth performance, serum parameters, and Cd concentration) were calculated to determine the representative module for further analysis. The top 10 genes with intramodular connectivity in their corresponding modules were identified as hub genes.

### 2.7. Statistical Analysis

All the data were subjected to a normal distribution test using SPSS 25 (IBM Corp, NY, USA). Differences between males and females within each group were compared using PERMANOVA (based on Bray–Curtis distance) vegan R package [25]. Growth performances and Cd concentration were normally distributed and were analyzed using an independent samples t test without the effect of gender and the interaction between gender and Cd. The serum parameters were analyzed using covariance analysis, with the data of Day 0 defined as covariates. Differences among groups were statistically assessed using Kruskal–Wallis for alpha diversity and PERMANOVA (based on Bray–Curtis distances) for beta diversity in QIIME2. *FDR* or *p* < 0.05 was considered statistically significant.

## 3. Results

### 3.1. Growth Performance, Morphology, and Serum Parameters

**Figure 1:**
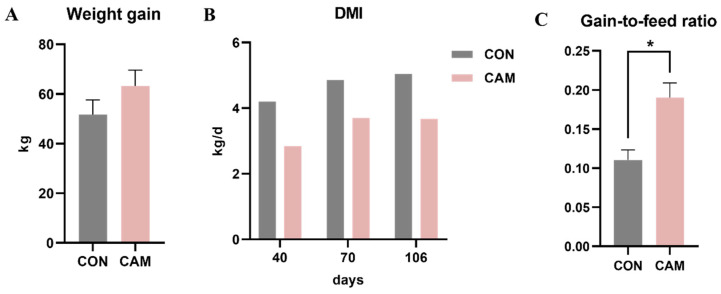
Weight gain (**A**), DMI on Day 40, 70, and 106 (**B**), and gain-to-feed ratio (**C**). * represents *p* < 0.05.

**Figure 2:**
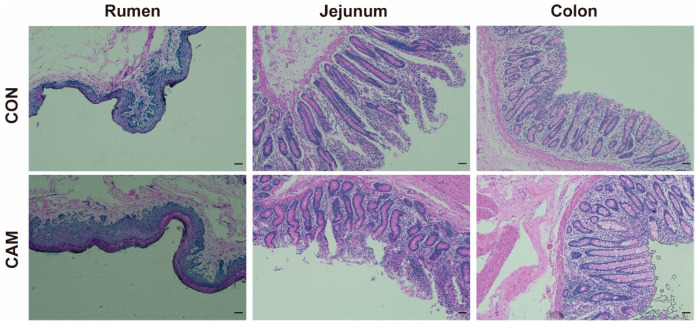
Photomicrograph of rumen, jejunum, and colon of cattle in CON and CAM. Scale bar: 50 μm. CON = control group. CAM = cadmium-accumulated maize group.

Weight gain, DMI, and gain-to-feed ratio were presented in Figure 1. Analysis of these data revealed that feeding Cd-accumulated maize silage had no adverse effect on weight gain, while the DMI of CAM cattle was lower than CON cattle, and CAM cattle exhibited a higher feed conversion efficiency (higher gain-to-feed ratio). For morphological observation, no histopathological damage and obvious difference were observed in the rumen, jejunum, and colon between the CON and CAM cattle (Figure 2). Based on our previous study [13], it was observed that cattle fed with Cd-accumulated maize silage displayed a higher (*p* < 0.05) levels of BUN, serum IgA, IgG, C3, C4, LDL-C, and CHOL and decreased serum IL-6 and LPS (Figures S1 and S2).

### 3.2. Cadmium Concentration in Blood and in Gastrointestinal Luminal Contents and Tissues

**Figure 3:**
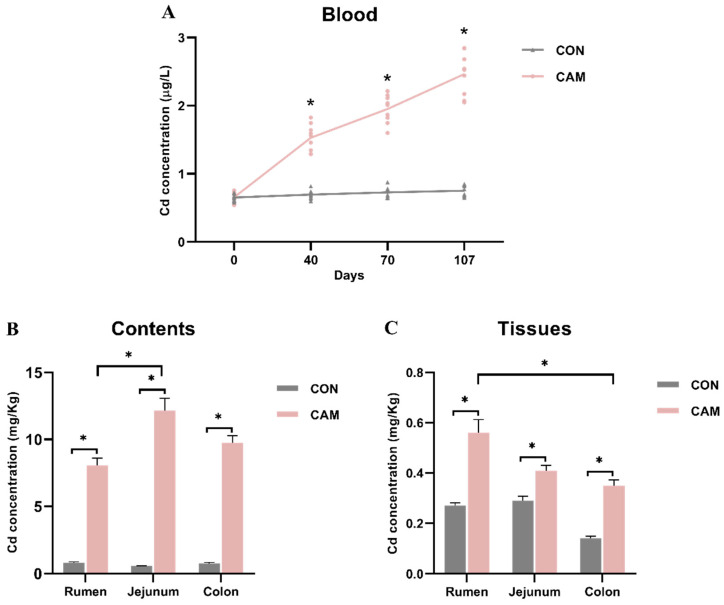
Cd concentrations in blood (**A**) of CON and CAM cattle during the feeding trial, and in luminal contents (**B**) and tissues (**C**) of the rumen, jejunum, and colon of CON and CAM cattle. * represents *p* < 0.05.

Concentrations of Cd are presented in Figure 3. Analysis of blood samples revealed that Cd concentrations in CAM cattle exhibited a gradual increase over time and were significantly higher than those in CON cattle from Day 40 (Figure 3A). The Cd concentrations from rumen to colon showed that Cd not only existed in the luminal contents of the gastrointestinal tract but could also be detected in the tissues (Figure 3B,C). In the CON, Cd concentration in the luminal content along the gastrointestinal tract remained stable (Figure 3B), and the Cd concentration in the jejunum tissue was similar to that in the rumen and approximately two times higher than that in the colon (Figure 3C). In the CAM, the jejunum contents had the highest Cd concentration (12.152 mg/kg), followed by the colon with 9.763 mg/kg and rumen with 8.072 mg/kg (Figure 3B). However, in the tissue, the highest Cd concentration was observed in the rumen (0.558 mg/kg), followed by the jejunum with 0.412 mg/kg and colon with 0.345 mg/kg (Figure 3C).

### 3.3. Profiles of Gastrointestinal Bacteria

**Figure 4:**
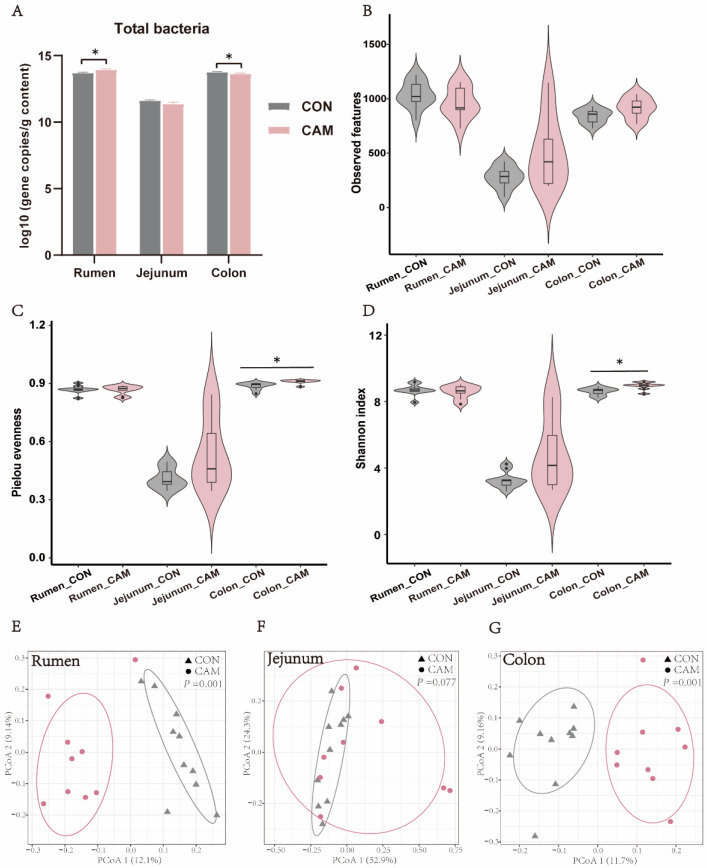
Effect of feeding Cd-accumulated maize silage on microbiota in luminal contents of rumen, jejunum, and colon. (**A**) The total population of bacteria. (**B**–**D**) Alpha diversity (observed features, Pielous evenness, and Shannon index) in different gastrointestinal segments of CON and CAM, respectively. (**E**–**G**) Beta diversity of the cattle in the rumen, jejunum, and colon. * represents *p* < 0.05.

The principal coordinates analysis revealed that there was no significant difference between male and female individuals within the same group (except for the colon of CON cattle) (Figure S3); thus, the effects of gender were not considered in this study. The total bacterial population (16S rRNA gene copies/g content sample) was significantly higher in the CAM rumen (*p* < 0.05) than that in the CON rumen, and was significantly lower (*p* < 0.05) in the CAM colon compared to the CON colon, and there was no significant difference in the jejunum between the CON and CAM (Figure 4A). For sequencing, a total of 5,430,869 quality-filtered amplicon sequences (accession number: PRJNA933805) were obtained for the 57 content samples with an average of 95,275 ± 23,022 sequences per sample: ten samples each from CON in the rumen (77,886 ± 10,072), jejunum (123,339 ± 115,17), and colon (88,612 ± 13,096) and nine samples each from CAM in rumen (68,950 ± 3946), jejunum (120,692 ± 15,770), and colon (91,747 ± 7170). The sequencing depth coverage reached over 99.60% on average (98.99% to 99.98%). In the luminal contents of rumen, jejunum, and colon of the CON cattle, an average of 1038, 279, and 839 observed features per sample were identified, with a Shannon diversity index of 8.70, 3.28, and 8.61 in the rumen, jejunum, and colon, respectively (Figure 4B,D). In the CAM, the observed features changed to 964, 522, and 916, and the Shannon diversity changed to 8.59, 4.85, and 8.94 in rumen, jejunum, and colon, respectively (Figure 4B,D). The Pielou evenness and Shannon diversity index of the bacterial community differed between the CON and CAM colons (*p* < 0.05), but not in the rumens and jejunums (*p* > 0.05, Figure 4C,D). The principal coordinates analysis of CON and CAM cattle showed no significant difference (*p* = 0.077) in the jejunum, but there were significant differences (*p* < 0.01) observed in the rumen and colon.

**Figure 5:**
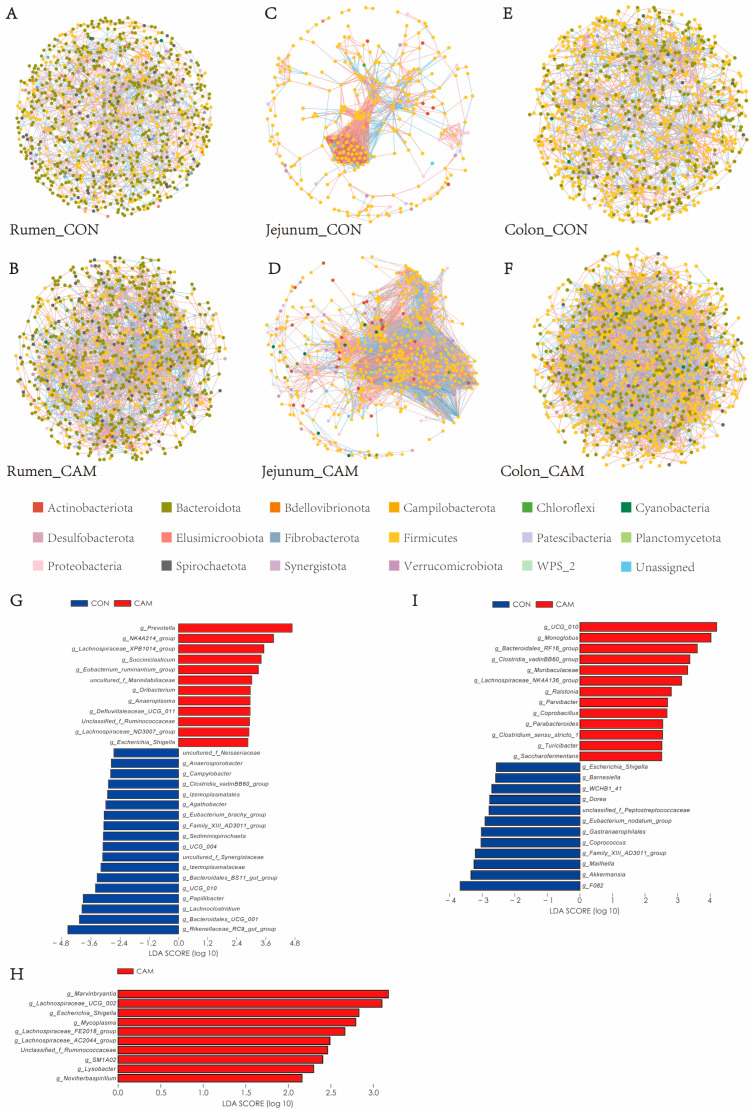
Effect of feeding Cd-accumulated maize silage on microbial networks and compositions in rumen, jejunum, and colon. (**A**–**F**) Networks of microbial interactions in the rumen, jejunum, and colon in CON cattle and CAM cattle, respectively. A node represents an ASV in the network. Red and blue lines represent positive and negative correlations, respectively. (**G**–**I**) The most differentially abundant genera in the rumen, jejunum, and colon between CON and CAM were identified using the LDA score, which was generated from LEfSe analysis.

The co-occurrence pattern for the rumen, jejunum, and colon in the CON cattle consisted of 975, 303, and 1013 nodes, respectively, with 2109, 1704, and 2140 edges (Figure 5A,C,E). In the CAM cattle, the number of nodes changed to 958, 587, and 1182, and the number of edges changed to 3222, 9065, and 5094 for the rumen, jejunum, and colon, respectively (Figure 5B,D,F). Notably, the ASVs belonging to *Peptostreptococcaceae* (a family belonging to the *Firmicutes* phylum) displayed a prominent relationship with other ASVs in the jejunum, and these ASVs exhibited a strongly negative correlation with ASVs belonging to other families.

In the rumen, Cd-accumulated maize exposure resulted in the enrichment of several bacterial taxa in the CAM cattle, including *Prevotella*, *NK4A214* group, *Lachnospiraceae XPB1014* group, *Succiniclasticum*, *Eubacterium ruminantium group*, uncultured *Marinilabiliaceae*, *Oribacterium*, *Anaeroplasma*, *Defluviitaleaceae UCG-011*, *Lachnospiraceae ND3007* group, *Escherichia Shigella*, and unclassified genera within *Ruminococcaceae* (Figure 5G). However, the *Rikenellaceae RC9 gut* group, *Bacteroidales UCG-001*, *Lachnoclostridium*, *Papillibacter*, *Oscillospirales UCG-010*, *Bacteroidales BS11 gut* group, *Izemoplasmataceae*, uncultured *Synergistaceae*, *Erysipelatoclostridiaceae UCG-004*, *Sediminispirochaetam*, *Family XIII AD3011* group, *Eubacterium brachy* group, *Agathobacter*, *Izemoplasmatales*, *Clostridia vadinBB60* group, *Campylobacter*, *Anaerosporobacter*, and uncultured *Neisseriaceae* were impoverished in the rumen of CAM cattle (Figure 5G). In the jejunum, *Marvinbryantia*, *Lachnospiraceae UCG-002*, *Mycoplasma*, *Escherichia Shigella*, *Lachnospiraceae FE2018* group, *Lachnospiraceae AC2044* group, unclassified genera belonging to *Ruminococcaceae*, *SM1A02*, *Lysobacter*, and *Noviherbaspirillum* were enhanced in the CAM cattle (Figure 5H). In the colon, *Oscillospirales UCG-010*, *Monoglobus*, *Bacteroidales RF16* group, *Clostridia vadinBB60* group, *Muribaculaceae*, *Lachnospiraceae NK4A136* group, *Ralstonia*, *Parvibacter*, *Coprobacillus*, *Parabacteroides*, *Clostridium sensu stricto 1*, *Turicibacter*, and *Saccharofermentans* were enriched in the CAM cattle, while *F082*, *Akkermansia*, *Maihella*, *Barnesiella*, *Family XIII AD3011* group, *Coprococcus*, *Gastranaerophilales*, *Eubacterium_nodatum*_group, unclassified genera within *Peptostreptococcaceae*, *Dorea*, *WCHB1-41*, and *Escherichia Shigella* were depleted (Figure 5I).

### 3.4. Profiles of Gastrointestinal Transcriptome

**Figure 6:**
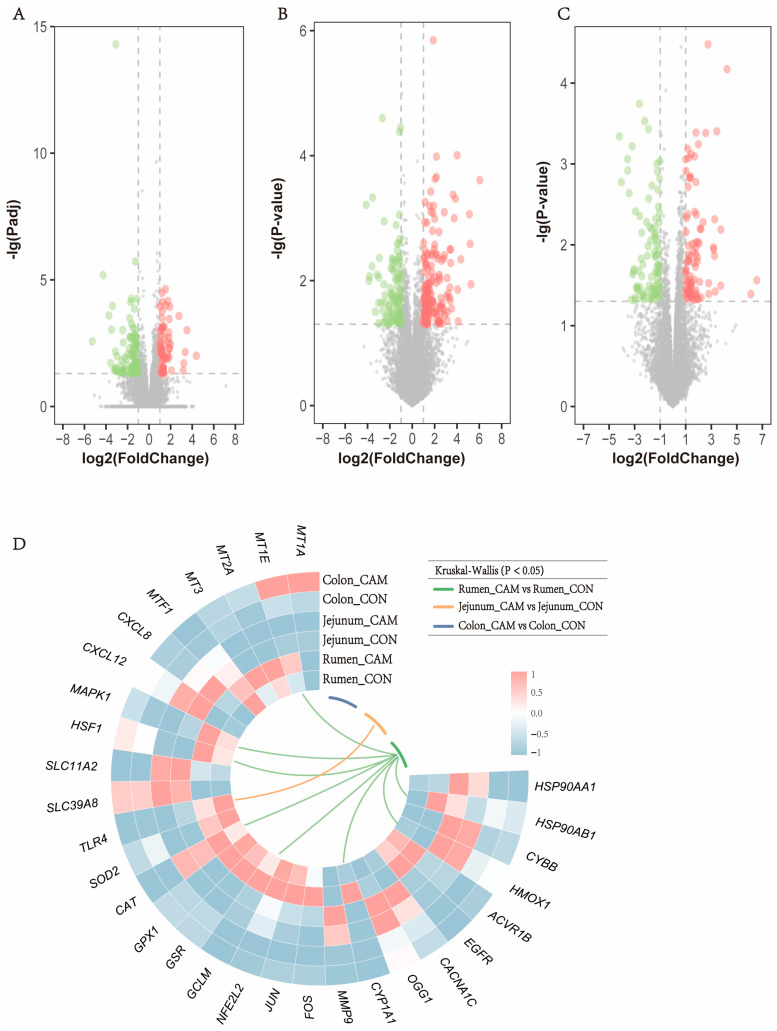
Effect of feeding Cd-accumulated maize silage on expressions of host genes in gastrointestinal tract. (**A**–**C**) Volcano plots display the differentially expressed genes of the rumen, jejunum, and colon. Red and green dots represent up regulated and down regulated differentially expressed genes, respectively. (**D**) Expression of cadmium-related genes in tissues in the three gastrointestinal segments.

Across all 54 tissue RNA samples, a total of 2691.2 million high-quality paired reads were obtained using RNA sequencing (accession number: PRJNA933098), with an average of 49.8 ± 8.6 million reads per sample: ten samples each from rumen-CON (46.7 ± 2.7) and jejunum-CON (61.7 ± 5.8), eight samples from colon-CON (46.0 ± 2.5), nine samples each from rumen-CAM (39.7 ± 3.4) and jejunum-CAM (56.7 ± 5.8), and eight samples from colon-CON (46.4 ± 4.3). In total, 17,258, 18,896, and 18,099 genes were identified as expressed genes in the rumen, jejunum, and colon, respectively. Furthermore, a total of 157, 253 and 174 DEGs between CON and CAM were identified in the rumen, jejunum, and colon, respectively (Figure 6A–C). The genes related to Cd transport and response can reflect the effects of Cd on the host gastrointestinal tract. The expression levels of these Cd-related genes are presented in a heatmap (Figure 6D). Compared to CON cattle, *MT1A* and *MT1E* were highly expressed in the colon of CAM cattle, while *MT2A* and *MT3* showed higher expression in the rumen of CAM cattle. Similar expression levels of genes related to oxidative stress and inflammation were observed in the CON and CAM cattle, although these genes fluctuated in different digestive tract tissues.

**Figure 7:**
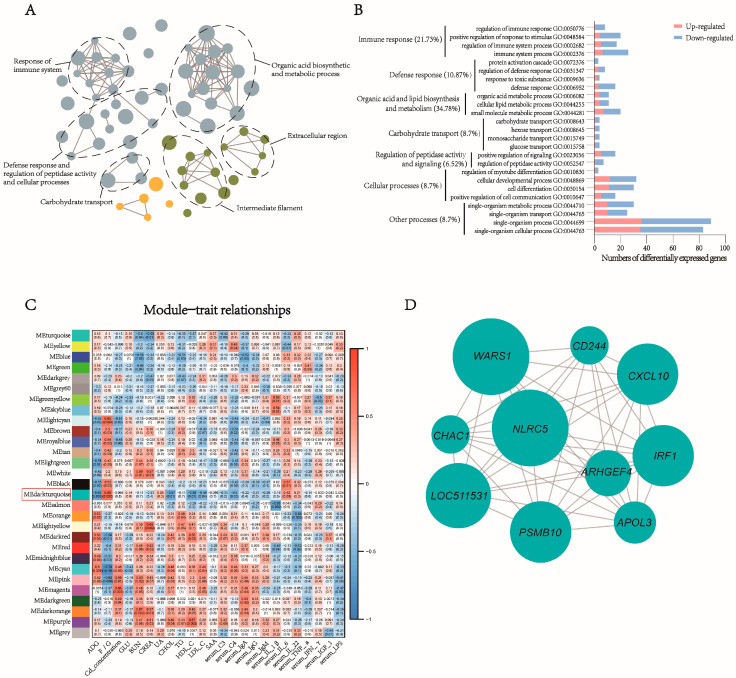
Gene ontology enrichment of differentially expressed genes and weighted coexpression network analysis in rumen of CON and CAM cattle. (**A**) Clusters of GO terms, including biological process (blue), molecular function (yellow), and cellular component (green). (**B**) Genes in representative biological process terms. (**C**) The relationships between coexpressed modules and performance. (**D**) Top 10 genes of connectivity in MEdarkturquoise; node size indicates intramodular connectivity.

In the rumen, the DEGs were significantly enriched in categories related to the response of immune system and defense, peptidase activity, and carbohydrate metabolism (Figure 7A). Organic acid and lipid biosynthesis and metabolism (34.78%), immune response (21.73%), defense response (10.87%), carbohydrate transport (8.7%), cellular processes (8.7%), regulation of peptidase activity and signaling (6.52%), and other processes (8.7%) were the major biological processes (Figure 7B). The DEGs of the jejunum and colon were primarily enriched in developmental process and peptidase activity (Figures S4A,B and S5A,B). Based on WGCNA, 29, 22, and 28 modules were identified in the coexpression networks of total genes in the rumen, jejunum, and colon, respectively (Figure 7C, Figures S4C and S5C). Among these modules, MEdarkturquoise (involved 165 genes) of rumen, MEtan (involved 1004 genes) of jejunum, and MElightpurple (involved 178 genes) of colon were associated with the most traits and were selected as representative module for the rumen, jejunum, and colon, respectively (Figure 7C, Figure S4C and S5C). MEdarkturquoise was significantly correlated (*R* > 0.5, *p* < 0.05) with average daily gain, feed conversion ratio, CHOL, HDL-C, LDL-C, and serum C4, MEtan was significantly correlated with BUN, CREA, TG, and HDL-C, and MElightpurple was significantly correlated with BUN, IgM, TNF-α, and LPS. The hub genes of MEdarkturquoise and MElightpurple were mainly involved in immune system regulation, such as *PSMB10*, *CD244*, *IRF1*, and *CXCL10* in MEdarkturquoise and *CD6*, *CD247*, *CD3D*, and *CD3E* in MElightpurple. Most of the hub genes of MEtan were ribosomal protein-encoding genes, including *RPL8*, *RPL10*, *RPS5*, and *RPS24* (Figure 7D, Figures S4D and S5D).

## 4. Discussion

Based on the weight gain and DMI, our findings indicated that feeding Cd-accumulated maize silage with a Cd concentration of 6.74 mg/kg DM could increase the feed utilization of cattle, consistent with previous studies that low-dose Cd exposure did not adversely affect animal growth and even promoted growth of animals at low doses [26,27]. The Cd concentration of CAM remained consistent during the experiment, and this might allow cattle to gradually establish the ability of tolerance to Cd. Future research is imperative for delving into the tolerance mechanisms exhibited by cattle following the consumption of Cd-accumulated maize. Furthermore, it is essential to investigate the relationship between Cd tolerance and feed efficiency. Cd exposure can cause inflammation and tissue damage in the jejunum and colon [5,28]. As an important site for ingestion, jejunum also plays a primary role in Cd absorption [29]. Different to other studies [4,5], there was no inflammation and intestinal damage observed in our study, and that might be associated with a decrease in serum LPS, a proinflammatory factor produced by Gram-negative bacteria, and could stimulate the production of IL-6, induce inflammatory, and alter intestinal permeability [30]. After ingestion of Cd-containing diet, most of Cd is excreted by urine and feces, a small part of Cd is absorbed in the blood and transported to the liver, kidney, and other organs via the blood circulation system and is retained in these organs [31,32]. Consequently, the levels of Cd in the blood of CAM cattle gradually increased with the duration of exposure. The different Cd concentrations in the luminal contents and tissues of the gastrointestinal tract indicate differences in Cd accumulation among the three digestive sites, and the varied Cd concentrations may modulate gene expression in the host. The highest Cd concentration in the rumen tissue compared to the intestine indicates that rumen is a major site of Cd absorption and accumulation in the gastrointestinal tract. Metallothioneins (MTs) play critical roles in Cd binding and storage in vertebrates [33], and an increase in metallothionein isoforms may be associated with Cd retention. Thus, the significantly higher expression level of *MT-1E* in the rumen of CAM cattle may explain the highest concentration of Cd in the rumen epithelium of CAM cattle.

Previous studies have demonstrated that Cd exposure could lead to a significant decrease in the populations of total bacteria in the feces of mice [28]. In our study, we found that the microbiota in rumen and colon were more sensitive to Cd compared to the jejunum. Interestingly, the trends of Cd effects on bacterial populations were reverse in rumen and colon. In contrast to the intestine, Cd had a positive effect on the growth of bacteria in the rumen. Gut microbiota can bioaccumulate heavy metals and accelerate their excretion, thereby mitigating the toxicity of heavy metals to the host [34]. However, gut microorganisms are one of the targets of Cd toxicity and are susceptible to the toxic effects of heavy metals, including Cd, which could affect the microbial growth and various enzymatic activities [34,35]. Both the rumen and the colon are rich in diverse microbiota, which may render microbiota at these sites particularly sensitive to the effects of Cd. Nevertheless, the Cd concentration in the rumen content (8.072 mg/kg) was lower than that in the colon content (9.763 mg/kg), and the microbiota in the rumen might potentially tolerate this lower Cd concentration in the rumen, and the Cd concentration in the colon might exceed the upper threshold of Cd tolerance for colon microbiota.

Microbial interactions are always present when the intestines are under healthy conditions, and exposure to heavy metals can disturb these interactions [36]. In this study, we found that Cd-accumulated maize silage enhanced bacterial networks in the rumen, jejunum, and colon, suggesting the improvement of microbial correlations, which might be the reason for the increased feed efficiency. *Peptostreptococcaceae*, a family belonging to the *Firmicutes* phylum and known for its involvement in the production of SCFAs and increased abundance in ulcerative colitis [37,38], exhibited strong connectivity in the microbial network in the jejunum of the CAM cattle, indicating that the interactions among SCFA-producing bacteria might be intensified in the jejunum, which might lead to an enhanced production of SCFAs. However, a limitation of our study is the absence of measurement of SCFA concentrations, and the effects of Cd on SCFAs productions and SCFA-related bacteria in the gut of ruminants need further investigation. Heavy metals can induce intestinal mucosal damage [5,11] and alter the microenvironment for microbes, changing their microbial structures [36]. However, in our study, the morphology of the rumen, jejunum, and colon of cattle did not change after ingestion of Cd-accumulated maize silage. Therefore, the alterations in microbial networks and structures might be attribute to the response of some Cd-sensitive bacteria, such as SCFA-related bacteria in our study.

*Prevotella*, *Lachnospiraceae ND3007* group, *Lachnospiraceae NK4A136* group, and *Clostridiales vadinBB60* group are SCFA-producing bacteria [39,40,41,42]. These bacteria play a significant role in promoting gut health by preventing inflammation and maintaining the homeostasis of the intestinal barrier [43,44,45]. Notably, the *Lachnospiraceae NK4A136* group is a potential butyrate producer with protective and anti-inflammatory effects [41,46,47], and the *Clostridiales vadinBB60* group is correlated with propionate production and urinary metabolites that may modulate the energy metabolism of the host [42,48]. The enrichment of these bacterial groups in the rumen and colon might suggest that Cd might affect immune regulation and energy metabolism in these gastrointestinal segments. Moreover, the higher feed efficiency observed in our study was consistent with a previous study [26,27], and the study results indicated that Cd might promote energy utilization and enhance gastrointestinal protection in cattle by affecting bacteria with specific functions indirectly, such as SCFA-producing bacteria, anti-inflammatory bacteria, or metabolism-related bacteria.

Cd exposure has been shown to elevate the levels of inflammatory cytokines, which can directly induce inflammation [5,49]. For instance, Benvenga et al. [50] demonstrated that Cd could cause histological alterations and increase expressions of *CXCL10* in the thyroid gland of mice. Similarly, Xie et al. [51] revealed that exposure to Cd down-regulated the expression level of *C3* in the anterior intestine of *Pelteobagrus fulvidraco*, indicating the inhibition of intestinal immune regulation. Consistent with these previous studies, our results showed that Cd down-regulated several immune-related genes in the rumen, including *C3*, *C1S*, *PSMB8*, *PSMB10*, *CXCL10*, *IL-18BP*, *IL-26*, and *TNFSF14*, suggesting the latent inhibition and dysfunction of immunity in the rumen tissue. *C1S* and *C3* are the essential components of complement system, participating in the recognition and activation of the classical activation pathway of the complement system [52]. Immunoproteasomes, a subset of proteasomes which is highly expressed in the immune cells, perform various functions in immune regulation, including antigen presentation, differentiation of T-cell and B-cell, and activation of monocytic and dendritic cell [53]. *PSMB8* and PSMB10 are genes that encode protein subunit of the immunoproteasome complex [54,55]. The decreased expression levels of *C3*, *C1S*, *PSMB8*, and *PSMB10* in the rumen of CAM cattle indicated that long-term feeding of Cd-containing diet might induce the suppressive effects on immune regulation in rumen tissue. Similar to Xie et al. [51], our study showed that several pro-inflammatory cytokines (such as *CXCL10*, *IL-26*, and *TNFSF14*) were down-regulated in the rumen of CAM cattle, which indicated that ingested Cd might inhibit the immune response in the gastrointestinal tract at the genetic level. Moreover, *IL-22RA2*, a natural antagonist of proinflammatory cytokines (IL-22), exhibited an up-regulated expression level in the jejunum of CAM cattle, suggesting the potential inhibition of inflammation in the jejunum, and this might be associated with the intensive microbial interactions among ASVs belonging to *Peptostreptococcaceae*, which are involved in the gut inflammatory response. In addition, the expression level of *TNFSF4*, a pro-inflammatory cytokine, was down-regulated in the colon, which was consistent with the up-regulation of anti-inflammatory bacteria (*Lachnospiraceae NK4A136* group) in the colon. These results indicated that ingested Cd might weaken the immunity by down-regulating the expression of immune-related genes, and this might be associated with microbial alterations in the microbiota composition of the gastrointestinal tract, particularly those bacteria linked to immune regulation. However, in our study, the effects of Cd on immune regulations were only investigated at the genetic level. More comprehensive studies are needed in the future to explore the Cd effects on the immune system of ruminants and the correlation between the gut microbiota and immune system under Cd exposure.

Based on WGCNA, we identified a representative module and ten hub genes for each segment of the gastrointestinal tract. The hub genes in MEdarkturquoise and MElighterpurple were primarily related to immunology, including *PSMB10*, *CD244*, *IRF1*, and *CXCL10* in MEdarkturquoise, which were involved in antigen presentation, immune cell regulation, antiviral action, and immune activation, respectively [54,56,57,58]. Meanwhile, the hub genes of MElightpurple, *CD6*, *CD247*, *CD3D*, and *CD3E*, contributed to T-cell activation, thymocyte differentiation. and T-cell development [59,60,61], suggesting that the immune system might be a potential target of Cd toxicity in cattle, and Cd might affect immune regulation through modulating the expressions of immune-related genes. Although the functional enrichment analysis did not reveal immune-related terms or pathways enriched by DEGs in the colon, these hub genes in MElightpurple and MEdarkturquoise could imply that ingested Cd might induce potential effects on the gastrointestinal immune system. Ribosomal protein genes have ubiquitous expression across various cell types, and their products play a vital role in ribosome synthesis, which is crucial for cell growth and proliferation [62]. However, the association of ribosomal proteins with heavy metals exposure has been poorly studied. Given the significance of ribosomal proteins in cell growth and proliferation, it indicates that the hub genes in MEtan may play a pivotal role in the influence of Cd on intestinal development, which could be a novel perspective for further research.

## 5. Conclusions

In this study, we found that the ingestion of Cd-accumulated maize silage increased the gain-to-feed ratio without changing the morphology of the rumen, jejunum, and colon, increased the abundance of SCFA-related bacteria, and modulated gene expression levels in the rumen, jejunum, and colon of cattle. Our study suggested that feeding Cd-accumulated maize silage could promote feed utilization of cattle, which might attribute to the improvement of SCFA-producing bacteria and the enhancement of microbial interactions in the gastrointestinal tract. However, the transcriptome profiles revealed potential concerns regarding the long-term feeding of Cd-accumulated maize, which might cause gastrointestinal immune abnormality in cattle by down-regulating the immune-related genes. Our findings provided new insights into the biological effects of Cd on the gastrointestinal tract of ruminants and revealed that ruminants may have tolerance to dietary cadmium exposure. Furthermore, it would be interesting to explore their mechanisms in future research.

## Figures and Tables

**Table 1:** Ingredients and chemical composition of the total mixed ration administered to the cattle of CON and CAM.

Item	CON	CAM	SEM	*p*-Value
Ingredients (fresh basis, %)				
Straw	2.84	2.84	-	-
Corn silage	85.82	85.82	-	-
Pelleted concentrate ^1^	11.34	11.34	-	-
Total	100	100	-	-
Chemical composition ^2^ (DM basis, %)				
GE, MJ/kg	12.00	13.36	0.406	0.09
CP	10.79	11.45	0.292	0.30
EE	11.00	13.00	0.730	0.20
NDF	49.33	50.67	1.732	0.74
ADF	25.00	25.33	1.195	0.91
Ca	0.62	0.62	0.028	0.95
P	0.42	0.37	0.022	0.32
Cd, mg/kg	0.72	6.74	1.516	<0.01

CON = control group and CAM = Cd-accumulated maize group. ^1^ Pelleted concentrate was purchased from Purina with the following chemical composition (fresh basis, %): crude protein ≥ 20.0, crude fiber ≤ 10.0, ash ≤ 10.0, 0.5 ≤ Ca ≤ 1.5, *p* ≥ 0.45, 0.25 ≤ sodium chloride ≤ 1.0, and Lys ≥ 1. ^2^ GE = gross energy; DM = dry matter; CP = crude protein; EE = ether extract; NDF = neutral detergent fiber; ADF = acid detergent fiber; Ca = calcium; P = phosphorus; and Cd = cadmium.

## Data Availability

The datasets presented in this study can be found in online repositories. The names of the repository and accession numbers are as follows: NCBI Sequence Read Archive (https://www.ncbi.nim.nih.gov/sra) (accessed on 30 August 2023) and PRJNA933098 and PRJNA933805.

## Supplementary Materials

The following supporting information can be downloaded at: https://www.mdpi.com/article/10.3390/ani13193104/s1, Figure S1. Levels of blood biochemical parameters of cattle in CON and CAM. A-H: Glucose, blood urea nitrogen, creatinine, uric acid, cholesterol, triglycerides, high-density lipoprotein cholesterol and low-density lipoprotein cholesterol, * represents *p* < 0.05. Figure S2. Levels of blood immunological and inflammatory indexes of cattle in CON and CAM. A-H: Complement 3, complement 4, immunoglobulin A, immunoglobulin G, immunoglobulin M, interleukin-1β, interleukin-6, interleukin-22, interferon-γ, tumor necrosis factor-α, insulin growth gactor-1 and lipopolysaccharide, respectively. * represents *p* < 0.05. Figure S3. Principal coordinates analysis plot of rumen, jejunum and colon bacteria between CON and CAM cattle; M and F represents male and female, respectively. Figure S4. Gene ontology enrichment of differentially expressed genes and weighted coexpression network analysis in the jejunum of CON and CAM cattle. (A) Clusters of GO terms, including biological process (blue), molecular function (yellow) and cellular component (green). (B) Genes in representative biological process terms. (C) The relationships between coexpressed modules and performance. (D) Top 10 genes of connectivity in MEtan; node size indicates intramodular connectivity. Figure S5. Gene ontology enrichment of differentially expressed genes and weighted coexpression network analysis in the colon of CON and CAM cattle. (A) Clusters of GO terms, including biological process (blue), molecular function (yellow) and cellular component (green). (B) Genes in representative biological process terms. (C) The relationships between coexpressed modules and performance. (D) Top 10 genes of connectivity in MElightpurple; node size indicates intramodular connectivity.

## Abbreviations

ADF, acid detergent fiber; ASV, amplicon sequence variant; BUN, blood urea nitrogen; CAM, cadmium-accumulated maize group; CHOL, cholesterol; CON, control group; CP, crude protein; CREA, creatinine; C3/4, complement 3/4; DEG, differentially expressed genes; DM, dry matter; EE, ether extract; FFA, free fatty acid; GE, gross energy; GLU, glucose; HDL-C, high-density lipoprotein cholesterol; IFN-γ, interferon-γ; IgA/G/M, immunoglobulin A/G/M; IGF-1, insulin growth factor-1; IL-1β/6/22, interleukin-1β/6/22; LDL-C, low-density lipoprotein cholesterol; LPS, lipopolysaccharide; MT, metallothionein; NDF, neutral detergent fiber; ROS, reactive oxygen species; TG, triglycerides; SAA, serum amyloid-A; SCFAs, short-chain fatty acids; TNF-α, tumor necrosis factor-α; and UA, uric acid.
