# Supplementary material for: Assessing the Effects of Dietary Cadmium Exposure on the Gastrointestinal Tract of Beef Cattle via Microbiota and Transcriptome Profile
Source: Animals (Basel). 2023 Oct 5;13(19):3104. doi: 10.3390/ani13193104 (PMC10571678; doi:10.3390/ani13193104)
Supplement: Supplementary file 1 [file animals-13-03104-s001.zip › animals-2641857-supplementary.pdf]

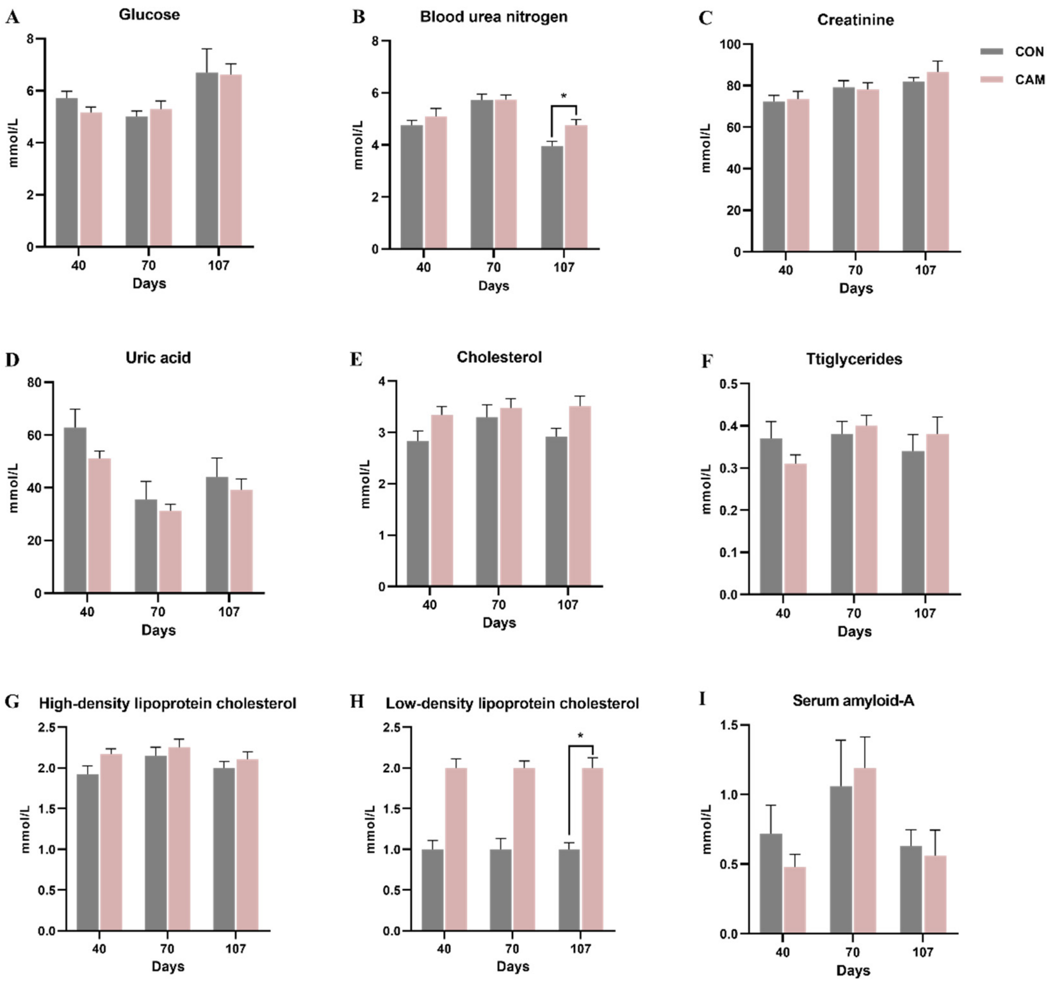

Figure S1. Levels of blood biochemical parameters of cattle in CON and CAM. A-H: Glucose, blood urea nitrogen, creatinine, uric acid, cholesterol, triglycerides, high-density lipoprotein cholesterol and low-density lipoprotein cholesterol, * represents $P < 0.05$. Data was cited from https://doi.org/10.1016/j.aninu.2023.06.012.

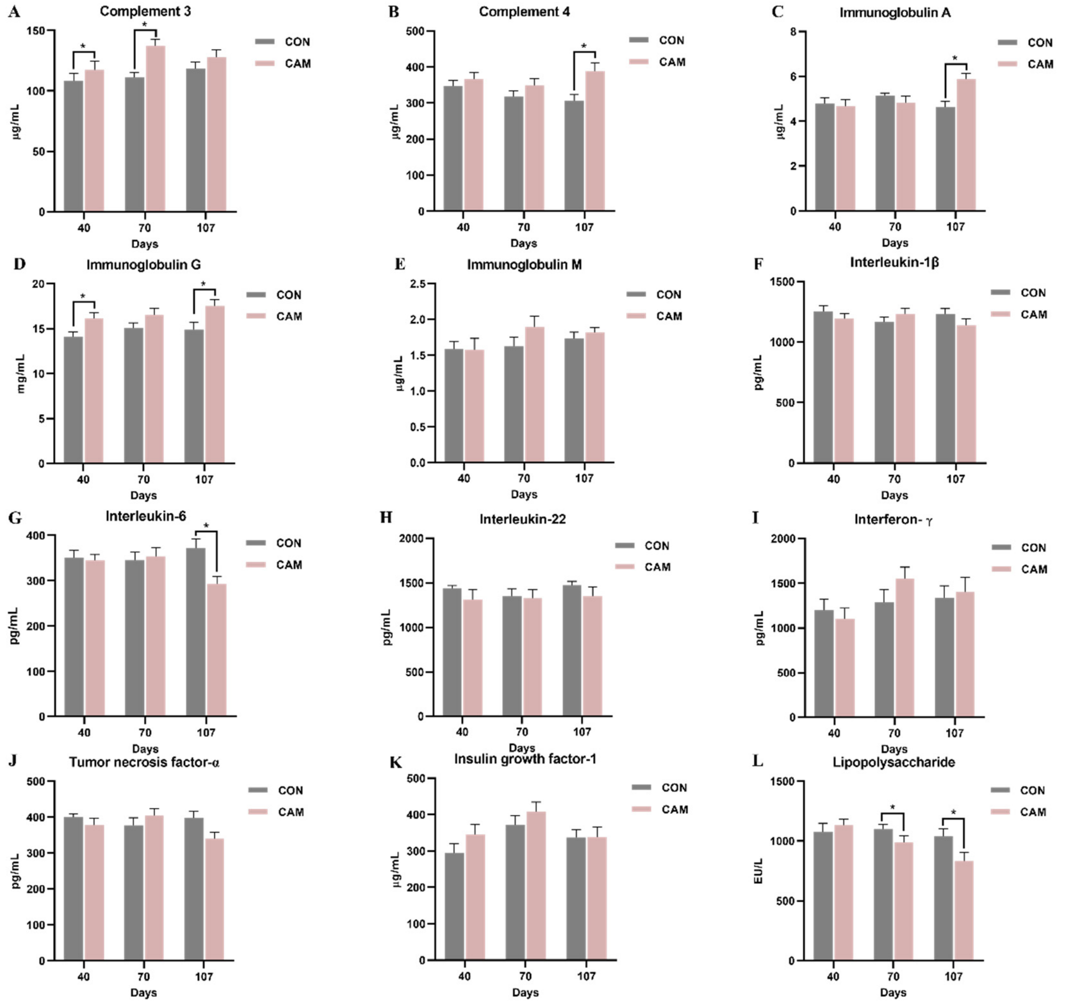

Figure S2. Levels of blood immunological and inflammatory indexes of cattle in CON and CAM. A-H: Complement 3, complement 4, immunoglobulin A, immunoglobulin G, immunoglobulin M, interleukin-1β, interleukin-6, interleukin-22, interferon-γ, tumor necrosis factor-α, insulin growth gactor-1 and lipopolysaccharide, respectively. * represents $P < 0.05$. Data was cited from https://doi.org/10.1016/j.aninu.2023.06.012.

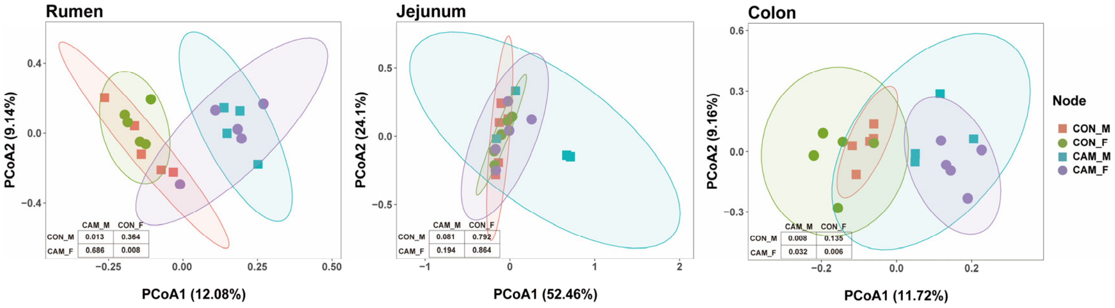

Figure S3. Principal coordinates analysis plot of rumen, jejunum and colon bacteria between CON and CAM cattle; M and F represents male and female, respectively.

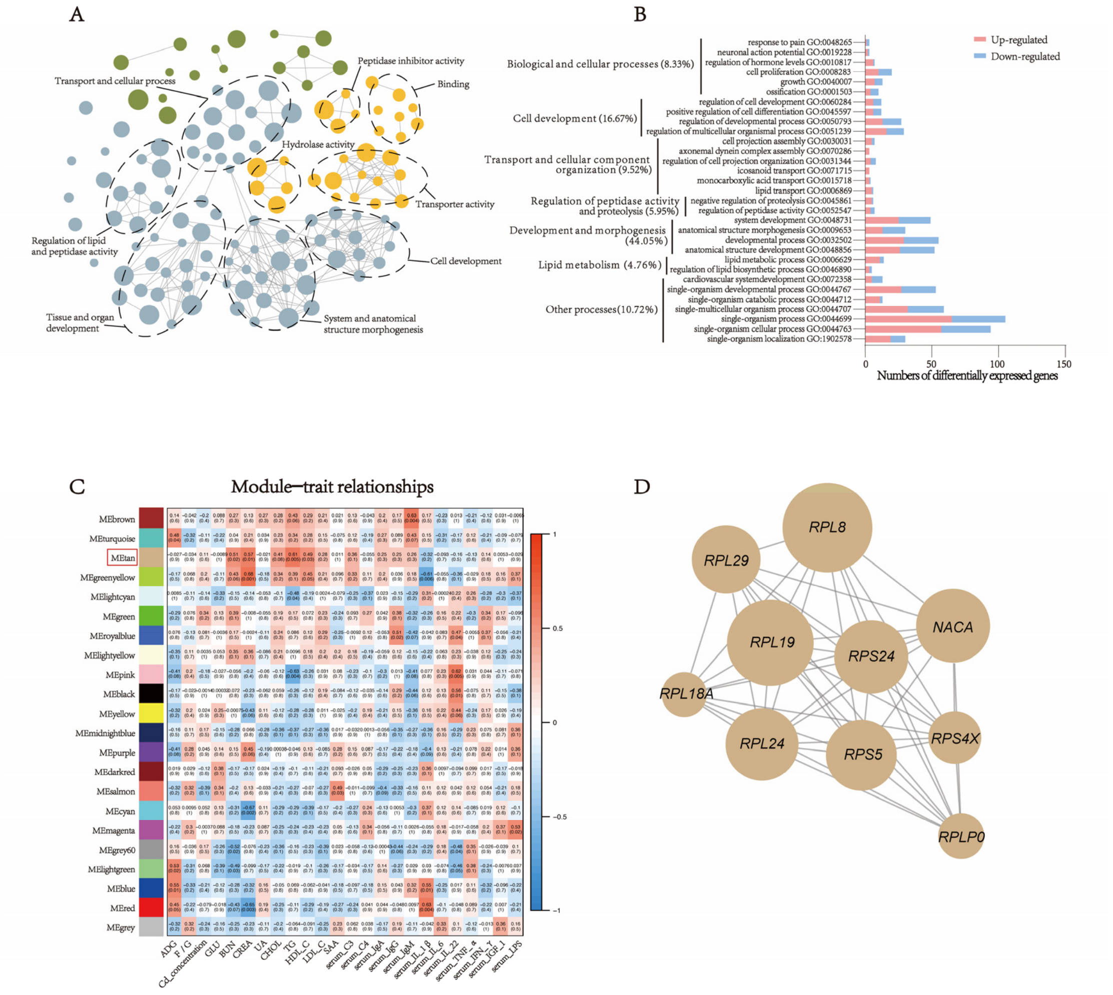

Figure S4. Gene ontology enrichment of differentially expressed genes and weighted coexpression network analysis in the jejunum of CON and CAM cattle. (A) Clusters of GO terms, including biological process (blue), molecular function (yellow) and cellular component (green). (B) Genes in representative biological process terms. (C) The relationships between coexpressed modules and performance. (D) Top 10 genes of connectivity in MEtan; node size indicates intramodular connectivity.

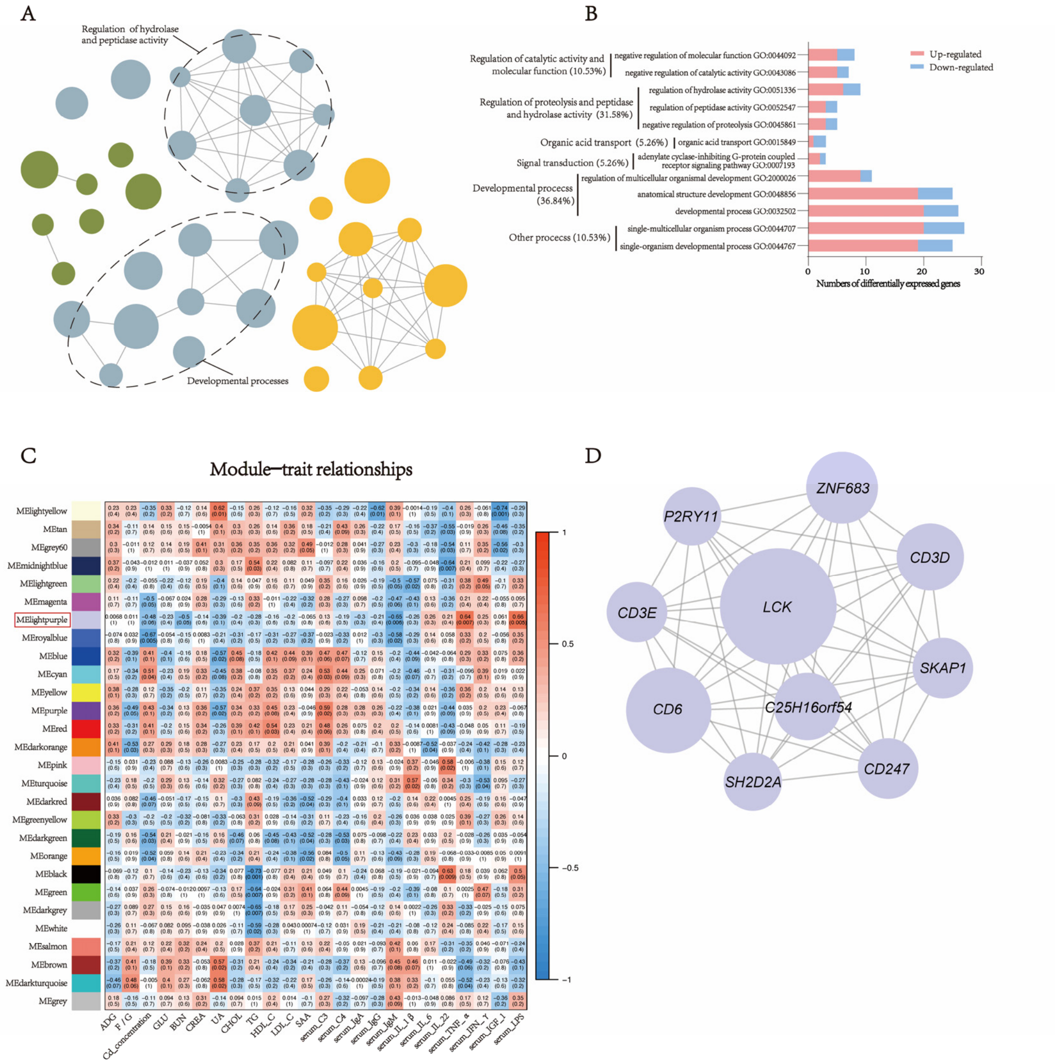

Figure S5. Gene ontology enrichment of differentially expressed genes and weighted coexpression network analysis in the colon of CON and CAM cattle. (A) Clusters of GO terms, including biological process (blue), molecular function (yellow) and cellular component (green). (B) Genes in representative biological process terms. (C) The relationships between coexpressed modules and performance. (D) Top 10 genes of connectivity in MElightpurple; node size indicates intramodular connectivity.
